# Chylopericardium following esophagectomy: a case report and systematic review

**DOI:** 10.1186/s13019-024-02536-x

**Published:** 2024-02-03

**Authors:** Xinglin Yang, Jinghong Zhang, Pengxia Sun, Jihai Liu, Jiangshan Wang, Huadong Zhu

**Affiliations:** 1 Department of Cardiology, Peking Union Medical College Hospital, Chinese Academy of Medical Sciences & Peking Union Medical College, Beijing, 100730 China; 2 Central Clinical School, Monash University, Melbourne, VIC 3004 Australia; 3 Emergency Department, State Key Laboratory of Complex Severe and Rare Diseases, Peking Union Medical College Hospital, Chinese Academy of Medical Sciences & Peking Union Medical College, Beijing, 100730 China

**Keywords:** Chylopericardium, Esophagectomy, Cardiac tamponade, Thoracic duct, Case report

## Abstract

**Background:**

Chylopericardium is a rare condition characterized by the accumulation of chyle in the pericardial space. It is most commonly caused by thoracic duct injury. Chylopericardium following esophagectomy is extremely rare but can cause life-threatening complications. This report presents a case of chylopericardium post-esophagectomy, resulting in cardiac tamponade and cardiac arrest. A systematic literature review was also conducted to facilitate the understanding of this rare condition.

**Case presentation:**

A 41-year-old male was admitted to our hospital with intermediate to highly differentiated squamous cell carcinoma of the mid-thoracic esophagus (clinical T4NxM0). He underwent thoracoscopic-laparoscopic esophagectomy with cervical anastomosis. On postoperative day 1, patient had a cardiac arrest secondary to cardiac tamponade, requiring emergency ultrasound-guided drainage. The drained fluid was initially serous but became chylous after the administration of enteral nutritional emulsion. As a result of significant daily pericardial drainage, patient subsequently underwent thoracic duct ligation. The amount of drainage was substantially reduced post-thoracic duct ligation. Over a period of 2 years and 7 months, patient recovered well and tolerated full oral diet. A comprehensive literature review was conducted and 4 reported cases were identified. Among these cases, three patients developed pericardial tamponade secondary to chylopericardium post-esophagectomy.

**Conclusion:**

Chylopericardium is a rare but serious complication post-esophagectomy. Prompt echocardiography and thorough pericardial fluid analysis are crucial for diagnosis. Thoracic duct ligation has been shown to be an effective management approach for this condition.

## Background

Chylopericardium is a rare clinical condition characterized by a pericardial effusion composed of chyle from the lacteals via the thoracic duct. The etiology can be idiopathic or secondary to surgery, radiation exposure, trauma, malignancy, and infection [1].

Esophagectomy is the mainstay approach for managing esophageal cancer. It is associated with the potential development of cardiac complications, namely atrial fibrillation and myocardial infarction [2, 3]. Chylopericardium is extremely rare post-esophagectomy. The acute leakage of chyle post-esophagectomy can lead to rapid accumulation of pericardial fluid and increased intrapericardial pressures, resulting in cardiac tamponade. Nevertheless, early identification and prompt management of this condition poses certain challenges.

The incidence of chylopericardium post-esophagectomy is extremely low. As of our current understanding, no specific reports in the literature have identified its exact prevalence. Available literatures are only limited to case reports, which further highlights the rarity of this complication.

We presented a case of chylopericardium, which had led to cardiac tamponade and cardiac arrest, in the setting of esophagectomy. We also performed a systematical literature review on this rare condition.

## Case report

A 41-year-old male, who had clinical T4NxM0 intermediate to highly differentiated squamous cell carcinoma of the mid-thoracic esophagus, was managed by a multidisciplinary team consisting of surgeons, oncologists, radiologists, and dietitians. Patient underwent a combined thoracoscopic-laparoscopic esophagectomy with cervical anastomosis at Peking Union Medical College Hospital. Patient had no other comorbidities. According to the Eastern Cooperative Oncology Group (ECOG) scale, his performance status was rated as 1. The preoperative nutritional assessment showed a body mass index of 23.2 kg/m², and a serum albumin level of 37 g/L, suggestive of an adequate nutritional status. The operation involved a 3-stage approach under general anesthesia. A single-lumen endotracheal tube was utilized, and a bronchial blocker was deployed for lung isolation. The thoracic stage of the surgical procedure involved thoracoscopic mobilization of the esophagus and regional lymphadenectomy (right recurrent laryngeal nerve nodes, left recurrent laryngeal nerve nodes, upper thoracic paraesophageal lymph nodes, and subcarinal lymph nodes). Notably, thoracic duct ligation was not performed. During the abdominal stage, the gastric mobilization and tubularization were performed through laparoscopic surgery. Lymph node dissection was conducted along the celiac and gastric vessels. A jejunostomy catheter was inserted to facilitate postoperative enteral feeding. The cervical stage involved the mobilization of the cervical esophagus and the creation of an esophagogastric anastomosis. The anastomosis was routinely performed using a circular stapler (Panther Healthcare, Beijing, China). Cervical lymphadenectomy was also performed. Vital signs were stable intraoperatively. Histopathology revealed ypT3N0M0. R0 resection was successfully achieved. Patient was transferred to the intensive care unit postoperatively.

On postoperative day (POD) 1, about 19-hour after the surgery, patient experienced a sudden onset of hemodynamic instability. In retrospect, subtle signs such as a slight decline of blood pressure and increase of heart rate were observed in the early postoperative period, which might have signified the beginning of the complication. However, these symptoms were initially attributed to the normal postoperative course. Echocardiography revealed a large pericardial effusion, necessitating prompt intervention to address cardiac tamponade. Thus, preparations were made for acute pericardiocentesis and central venous catheter insertion. Unfortunately, patient deteriorated rapidly and went into cardiac arrest. Cardiopulmonary resuscitation (CPR) was promptly initiated, and the patient was resuscitated to sinus rhythm after 2 min of intensive CPR, with a palpable carotid artery beat. Emergency pericardiocentesis was then performed using the ultrasound-guided Seldinger technique via an apical approach. About 200 ml of sanguineous fluid was successfully drained from the pericardial space. Analysis of the pericardial fluid showed a white blood cell count of 13.56 × 10^9^/L with 2.25 × 10^9^/L lymphocytes. Both cultures and cytology were negative.

The pericardial drain was left for continuous drainage of the pericardial effusion. The drainage was initially serous, with an approximate volume of 1500 ml daily. On POD 3, patient was commenced on glucose administration via the jejunostomy catheter. On POD 5, a significant change of the drain was observed following the administration of enteral nutritional emulsion (TP). The fluid became milky in consistency. Upon further examination of the pericardial effusion, the triglyceride level was found to be 5.65 mmol/L and the total cholesterol level was 1.04 mmol/L, yielding a cholesterol to triglyceride ratio of less than 1. The presence of fat globules was reviewed through the Sudan III staining. These results confirmed the diagnosis of chylopericardium. Patient was subsequently kept nil by mouth. Intravenous fluids and electrolyte replacement were commenced.

Over the next 3 days, patient remained to have high-volume output in the pericardial drainage, ranging from 1050 ml to 1710 ml per day. On POD8, patient returned to operating theatre for a right-sided thoracoscopic thoracic duct ligation. The thoracic duct was dissected at the level of the inferior pulmonary vein, between the odd vein and the descending aorta. It was then double ligated with Hem-O-lock (Teleflex, Limerick, USA) in a low position. Pericardial fenestration was not performed because of the existence of a pericardial drainage tube.

Following the thoracic catheter ligation, patient’s pericardial drainage showed a significant reduction, with the daily amount decreasing to less than 100 ml. On POD 3 following the second surgery, the drainage tube was safely removed. Patient’s postoperative progression following the second operation was unremarkable, without the recurrence of cardiac tamponade or chyle leakage after resuming a regular diet. Patient was kept under observation for an extended period to monitor recovery, as well as receiving ongoing review by allied health including physiotherapists and dietitians. Patient was discharged home on POD31 of the first surgery.

Patient was followed up for a period of 2 years and 7 months post-surgery, during which he successfully managed his recovery at home, tolerating a full oral diet. The comprehensive follow-up protocol, which considered both physical and psychological aspects, included clinical evaluations every 3 months for the first year, then every 6 months thereafter. In addition to assessing cardiac function, nutritional status, and signs of recurrence of chylopericardium or malignancy through regular echocardiography, blood tests, and necessary imaging studies, psychological wellbeing was also monitored. This was particularly important due to the unexpected, life-threatening complications that initially led to episodes of anxiety and distress for the patient. However, with consistent psychological support from the healthcare team, including counseling and reassurance, patient gradually improved from the psychological perspective. Approximately 1-year post-surgery, the patient’s physical and mental states had essentially returned to baseline levels. Throughout the entire follow-up period, no signs of recurrent chylopericardium, cardiac tamponade, or cancer relapse were observed.

## Systematic review

A comprehensive systematic literature review was conducted to evaluate all available pertinent data. The methods of the analysis and inclusion criteria were specified in advance and documented in a protocol. Our review adheres to the Preferred Reporting Items for Systematic Reviews and Meta-Analyses (PRISMA) guidelines for systematic reviews [4]. Two authors (XY and JW) independently searched PubMed and EMBASE for relevant articles. The final search was completed on May 31st, 2023. The search string was (“chylopericardium” OR “chylous pericardial effusion” OR “chylopericardial tamponade”) AND (“esophagectomy” OR “oesophagectomy” OR “esophagus resection” OR “oesophagus resection”). The reference lists of all identified sources were reviewed for additional relevant articles.

**Fig. 1:**
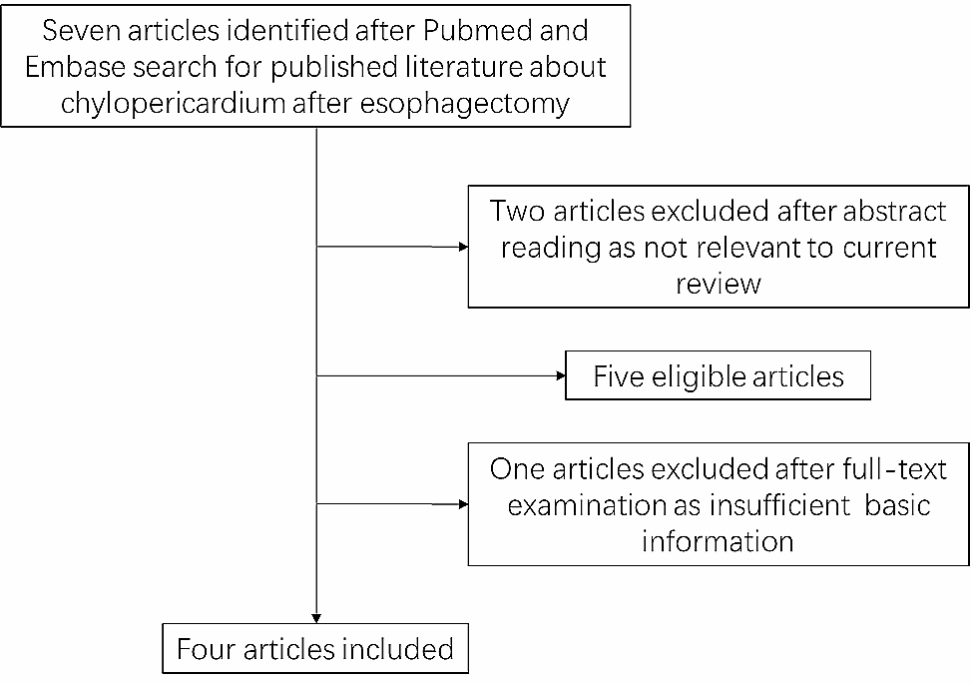
Flowchart illustrating the study identification and inclusion/exclusion process

The search procedure yielded a total of 7 references. Of these, 2 were excluded after abstract reading, as they were not relevant to the current review. One reference was excluded after full-text examination due to insufficient basic information. Hence, four publications, reporting 4 cases of chylopericardium post-esophagectomy, were selected [5–8]. A flowchart illustrating the study identification and inclusion/exclusion process was shown in Fig. 1. The search did not identify any randomized controlled trials or controlled cohort studies. It is worth noting that the first reported case of chylopericardium following esophagectomy was published in 1996 by Nakamura et al. in the journal of Surgical Today. This case report has been included in our systematic review. The epidemiological characteristics of all cases were summarized in Table 1. The median age was 51 years (range 41–75 years), with a 3:1 gender ratio (male:femal). The clinical features were reported in Table 2. Three of the patients underwent surgery for esophageal cancer, while one patient underwent surgery for high-grade dysplasia. It is worth noting that 3 out of the 4 patients were reported with pericardial tamponade. In terms of treatment, 2 patients received medical management, while 2 patients underwent thoracic duct ligation.

**Table 1:** Epidemiological characteristics of patients with chylopericardium following esophagectomy

Author	Number of patients	Age at onset (year)	Sex	Nationality
Nakamura S et al. 1996	1	57	Male	Japan
Stewart D et al. 2009	1	45	Female	UK
Kosugi SI et al. 2017	1	75	Male	Japan
Li L et al. 2020	1	41	Male	China

**Table 2:** Clinical features of patients with chylopericardium following esophagectomy

Author	Primary disease	Surgery	Symptoms	Color of effusion	Treatment	Follow-up
Nakamura S et al. 1996	Esophageal cancer (T3N1M0)	Radical esophagectomy with a three-field lymph node dissection involving a right thoracotomy and a partial median sternotomy	Palpitations	Milky	Pericardiocentesis, total fasting and intravenous hyperalimentation	eight months
Stewart D et al. 2009	High-grade dysplasia within a segment of Barrett’s metaplasia	Ivor-Lewis esophagogastrectomy	Cardiac tamponade	Chylous	Pericardiocentesis	Not reported
Kosugi SI et al. 2017	Esophageal cancer (T3N1M0)	Right transthoracic esophagectomy with extended mediastinal lymphadenectomy	Cardiac tamponade	From serous to chylous	Ligation of thoracic duct via left-sided thoracoscopic approach	Not reported
Li L et al. 2020	Esophageal cancer (T3N + M0)	Minimally invasive McKeown esophagectomy, three-field lymphadenectomy and jejunostomy	Cardiac tamponade	From serous to chylous	Ligation of thoracic duct via right-sided thoracoscopic approach	Not reported

## Discussion

Chylopericardium is a rare disorder that can occur either as a primary (idiopathic) condition or, more commonly, as a secondary condition resulting from injury to the thoracic duct [9]. The thoracic duct serves as a conduit for transporting chyle from the intestinal tract and closely passes by the pericardium as it travels from the cisterna chyli to the jugular and subclavian veins. Any disease capable of compressing, obstructing, or rupturing the thoracic duct and its branches can be the underlying cause of chylopericardium. The most common secondary causes include trauma, cardiac surgery, and congenital lymphangiomatosis [10]. However, chylopericardium following esophagectomy is an exceedingly rare occurrence, with only a few cases having been previously reported.

The pathogenesis of post-surgical chylopericardium is not entirely clear. The prevailing theory suggests that the primary mechanism involves a direct injury to the thoracic duct and mediastinal lymphatic vessels. This injury can result in the establishment of a communication between the lymphatic system and the pericardium, leading to the leakage of chyle into the pericardial cavity. Additionally, if there is an obstruction or disruption of the thoracic lymphatic ducts, this can lead to a significant increase in intraductal pressure, causing a reflux of lymphatic fluid back into the pericardial space [11]. In situations where chylopericardium is a consequence of direct injury, it may precipitate a specific point source leakage of chyle. On the other hand, if the condition ensues from obstructions that foster increased pressure, it may exhibit as diffuse leakage of chyle. In the instance of our patient who presented with sudden onset of postoperative chylopericardium, the condition was hypothesized to be associated with a localized surgical trauma. Lymphangiography can offer valuable insights into determining the cause, but regrettably, it is not a diagnostic procedure that our institution can provide. Furthermore, during the second surgery, the pericardium was not specifically examined in order to avoid further injury and that a further exploration would not change the management. Therefore, we are unable to ascertain the exact etiology of chylopericardium in this case.

The signs and symptoms of chylopericardium depend on the length of time over which pericardial fluid accumulates [12]. Chronic leakage of chyle is usually associated with systemic illnesses, such as malnutrition, metabolic derangements, and immunologic incompetence. However, acute leakage of chyle can lead to rapid pericardial fluid accumulation and increasing intrapericardial pressures, resulting in cardiac tamponade. This condition can be clinically manifested as Beck’s triad, which is composed of hypotension, distended neck veins, and distant heart sounds. These symptoms arise from decreased cardiac output due to impaired ventricular filling, augmented pressure during diastole that prevents blood from returning to the right atrium from the body circulation, and the insulating effect of the pericardial fluid, respectively. It is worth noting that 3 out of the 4 reported cases, in addition to our case, developed pericardial tamponade post-surgery. These patients deteriorated rapidly and urgent interventions were warranted. Therefore, the insidious disease onset and rapid clinical progression may represent clinical characteristics of chylopericardium in the setting of post-esophagectomy.

Chest X-ray, echocardiography, computed tomography, magnetic resonance imaging, and lymphangiography can reveal the presence of pericardial effusion [12]. Among these, echocardiography is the most important modality for the diagnosis and management of patients who develop or are clinically suspected to have cardiac tamponade. Chylopericardium is suggested by the identification of a milky effusion, which indicates its characteristic appearance, during pericardial fluid sampling. The definitive diagnosis is established through pericardial fluid analysis [12]. An increased level of triglyceride (typically exceeding 500 mg/dL (5.65 mmol/L)); and a cholesterol/triglyceride ratio of less than 1 confirms the diagnosis. Additionally, there should be no growth from the cultures and that lymphocyte should be predominant on cytology. Furthermore, the presence of fat globules can be observed on Sudan III staining in chylopericardium. Due to the requirement for preoperative fasting, the fluid nature of chylopericardium that occurs postoperatively can initially be clear and transparent. Among the five cases, including our case, three of them initially presented with serous pericardial effusion, which subsequently turned milky after administration of a nutrient solution containing fat. This phenomenon further complicates the diagnostic challenge for chylopericardium. Given the diagnostic challenges, lymphangiography plays a crucial role in the diagnosis of chylopericardium. This technique involves injecting a radiopaque contrast agent into the lymphatic vessels and using fluoroscopic images to trace the contrast as it moves through the lymphatic system [13]. Lymphangiography can also be utilized in combination with CT to demonstrate evidence of a fistulous connection between the thoracic duct and the pericardial sac [14]. However, it is worth noting that lymphangiography does require a higher level of technical expertise and equipment, and that it is not commonly used in practice. Therefore, the accessibility of lymphangiography may delay diagnosis of chylopericardium.

Once the diagnosis is confirmed, patients who are asymptomatic or mildly symptomatic can have an initial approach of treatment with dietary modifications without surgical intervention. The effusion may be controlled by adherence to a diet that is low in fat and high in medium chain triglycerides [1]. In favorable cases, the chylous effusion does not recur after a few weeks as result of dietary treatment that has diminished lymph flow and intra-lymphatic pressure [15]. However, patients with symptomatic effusion or a large uncontrolled effusion (defined as an average daily loss of chyle of 500 mL/day for 7 consecutive days) typically require invasive management including surgery [16].

Surgery typically involves ligation of the thoracic duct and tributary lymphatics, usually via a thoracic approach [12, 17]. Thoracic duct, particularly in the lower thoracic cavity, can be accessed surgically from either the right or the left side. In the lower thoracic cavity, the thoracic duct is situated between the azygos vein and the descending aorta. However, the heart and the descending aorta can obstruct the visibility of the duct on the left side [18]. It is therefore easier to approach the duct from the right hemithorax, as demonstrated in the cases reported by Li L and others, as well as our own case [8]. Nevertheless, the choice of surgical approach can be guided by different considerations. These include patients’ anatomy and their past surgical history, the location of lesions, as well as the expertise of surgeons. In the case presented by Kosugi SI et al., a left-sided approach was chosen [7]. This was likely due to the situation in which the fluid accumulation was bounded by the left pulmonary vein and left atrium, descending aorta, and vertebral column. Choosing a left-sided thoracoscopic approach allowed surgeons to directly address the site of chyle leakage in the posterior mediastinum, thereby enabling optimal visualization and management of the patient’s clinical condition. Suture ligation and clips have their merits in managing thoracic lymphatic leaks. Suture ligation, although technically demanding, is versatile and provides a stable closure for ducts of various shapes and sizes. However, it carries a risk of damaging surrounding tissues. Clips have limited applicability and carry a risk of postoperative dislodgement. The choice between the two largely depends on surgical preference, patient anatomy and suitability.

Pericardiotomy or pericardiectomy may also be considered in patients with persistent disease or in those who develop constrictive physiology, in order to secure stable drainage and prevent the onset of constrictive pericarditis [12, 19]. . Li L et al. did not proceed to pericardial interventions because of the pericardial drainage tube that was already inserted [8]. This was also the case in our patient. Kosugi et al. reported that a pericardial opening was created near the leakage point during esophagectomy [7]. This opening was subsequently used for drainage. The use of any sclerosing agents to minimize risk of recurrence, such as tetracycline or bleomycin, within the pericardial sac is contraindicated due to a high prevalence of post intervention constructive pericarditis [20].

The risk of recurrence for chylopericardium following esophagectomy remains largely undefined due to the condition’s rarity. A recently published meta-analysis incorporating data from 98 patients with chylopericardium presented a diverse range of etiologies [9]. In this meta-analysis, follow-up information was documented in 72% of all patients, with a mean follow-up duration of 180 days (first quartile [Q1] 90 days, third quartile [Q3] 377 days). Within this cohort, recurrence was observed in 16% (*n* = 16). Therefore, it is imperative to conduct vigilant monitoring of patients for potential recurrence signs. This includes regular echocardiography and rigorous nutritional assessment.

Chylopericardium is a rare but serious complication post-esophagectomy. A well-coordinated multidisciplinary approach, as demonstrated in our case, is essential for management. This involves surgeons performing the esophagectomy, cardiologists managing cardiac complications, radiologists providing crucial imaging and guidance, and dietitians ensuring postoperative nutritional care. Despite the rarity and lack of standardized protocols, our case report highlights the long-term effectiveness of thoracic duct ligation and the need for thorough diagnosis including prompt echocardiography and pericardial fluid analysis. The role of postoperative nutritional management and the reason for the occurrence of this complication after esophagectomy will need future research. Our case advocates the need for continued research in order to develop a guideline for this life-threatening postoperative complication.

## Conclusion

Chylopericardium is a rare but can be a serious complication post-esophagectomy. It requirs clinicians’ vigilance due to its subtle onset and rapid disease progression. Immediate echocardiography and thorough pericardial fluid analysis are the keys for diagnosis. This case report, with the longest follow-up currently available, affirms thoracic duct ligation as an effective long-term solution, especially when continuous drainage is achieved.

## Data Availability

Not applicable.

## Abbreviations

CPR: Cardiopulmonary resuscitation
CT: Computed tomography
ECOG: Eastern Cooperative Oncology Group
POD: On postoperative day
